# Ultrasound Diagnostic and Therapeutic Injections of the Hip and Groin

**DOI:** 10.5334/jbr-btr.1371

**Published:** 2017-12-16

**Authors:** Phey Ming Yeap, Philip Robinson

**Affiliations:** 1 Musculoskeletal Radiology, Radiology Department, Leeds Teaching Hospitals, Chapel Allerton Hospital, Leeds, UK; 2 University of Leeds and NIHR Leeds Musculoskeletal Biomedical Research Centre, Chapel Allerton Hospital, Leeds, UK

**Keywords:** Ultrasound, Diagnostic, Therapeutic, Injection, Hip, Groin

## Abstract

Hip and groin pain often presents a diagnostic and therapeutic challenge. The differential diagnosis is extensive, comprising intra-articular and extra-articular pathology and referred pain from lumbar spine, knee and elsewhere in the pelvis. Various ultrasound-guided techniques have been described in the hip and groin region for diagnostic and therapeutic purposes. Ultrasound has many advantages over other imaging modalities, including portability, lack of ionising radiation and real-time visualisation of soft tissues and neurovascular structures. Many studies have demonstrated the safety, accuracy and efficacy of ultrasound-guided techniques, although there is lack of standardisation regarding the injectates used and long-term benefit remains uncertain.

## Introduction

The hip and groin are sites of multiple injuries and inflammatory conditions, including intra-articular and extra-articular pathology, giving rise to an extensive differential diagnosis for hip and groin pain [1,2]. Pain originating from different anatomical areas such as lumbar spine, knee and pelvis can also be referred to the hip and groin. Often, patients with hip conditions have concomitant knee or spine conditions, which may present difficult therapeutic and diagnostic dilemmas [3].

Given the complexity of hip and groin anatomy and clinical conditions, imaging-guided injections are useful both for the diagnostic workup and treatment [4]. The main advantages of ultrasound-guided injection are its safety, portability and lack of ionising radiation. Injectates can include corticosteroid, local anaesthetic, platelet-rich plasma (PRP), viscosupplement and dextrose prolotherapy.

This article reviews the commonly performed diagnostic and therapeutic ultrasound-guided injections of the hip and groin region, specifically focusing on the anterior structures including the hip joint, iliopsoas, greater trochanter, pubic symphysis and lateral cutaneous nerve. The anatomy, indication, accuracy and efficacy of these procedures, along with the potential approaches are addressed.

## General Principles for Ultrasound-guided Injection

For all ultrasound-guided hip and groin procedures, the usual standards for musculoskeletal interventional procedures apply (e.g. review of previous imaging, informed consent and appropriate local anaesthetic).

The choice of ultrasound probe is important – use of a high-frequency (> 10 MHz) linear array transducer is recommended but lower-frequency curvilinear probes may be occasionally required to visualise deep structures in larger patients. A preliminary diagnostic sonographic examination, including colour Doppler of the area to be punctured is necessary to define the relationship of adjacent neurovascular structures. The full description of how to perform ultrasound assessment of the hip and groin region is however beyond the scope of this article [5].

Injections should be performed with adherence to aseptic technique although this varies between institutions and radiologists attributable to resources, training, perceived risk and experience [6]. In a survey of 250 health professionals in the United Kingdom, 43.5% believed infection rates were < 1/1000 following intra-articular injections, 33.0% perceived rates were < 1/100, and 2.6% perceived the risk as negligible [7]. Sterile preparation of the entire injection field, including adjacent skin where the gel and probe are applied, is recommended [6]. Areas of superficial infection such as cellulitis or abscess should be avoided to prevent deeper spread.

**Figure 1:**
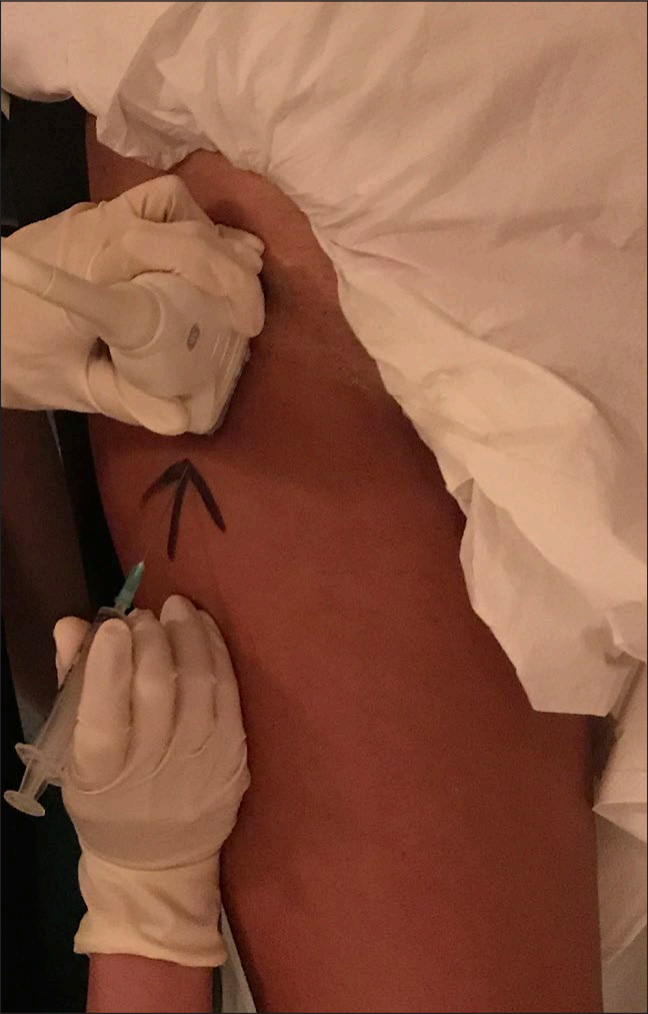
Anterior longitudinal approach for an in-plane hip joint injection. An arrow parallel to the long axis of the transducer is drawn on the skin adjacent to the end of transducer where the needle will be introduced.

After planning a safe route of access, a line parallel to the long axis of the transducer is drawn on the skin adjacent to the end of transducer where the needle will be introduced. Once the patient’s skin is sterilised and initial needle entry is made adjacent to the mark, the probe can be returned quickly to the same location and orientation by aligning to the skin mark (Figure 1). The needle is directed toward the intended target by a freehand technique. The needle size, length and type should be selected based on the site, depth and patient’s body habitus. 22–24G needles are sufficed for most injections.

## Ultrasound-guided Injection for Specific Anatomic Hip and Groin Region: Anatomy, Indication, Supporting Evidence and Injection Approach

### Hip joint

The hip joint is a ball-and-socket synovial joint, formed by an articulation between the femur and acetabulum. Intra-articular aetiology of hip pain includes osteoarthritis, inflammatory arthropathy, acetabular labral tears and femoro-acetabular impingement. Injection of short and long acting anaesthetic agents can be useful in confirming hip pathology and differentiating asymptomatic intra-articular pathology from extra-articular conditions that may be the source of symptoms. Complete relief of hip pain following intra-articular injection of local anaesthetic is associated with good surgical outcome following joint replacement [8].

Initial treatment options include activity modification, analgesia and physical therapy. When symptoms persist despite these measures, hip injections can be considered. Intra-articular hip injections can be technically challenging due to depth, variable body habitus, and the proximity to the femoral neurovascular bundle. Image guidance is therefore advocated to ensure safe and accurate needle placement [9]. Fluoroscopic-guidance was the mainstay imaging-guidance hip injection, but ultrasound-guidance is becoming increasingly prevalent due to its accuracy with visualisation of soft tissue and neurovascular structures, less associated cost and no ionising radiation exposure or risk of contrast agent reactions [10]. A position statement by the American Medical Society for Sports Medicine reviewed the literature and found several level one studies of ultrasound guided hip injections with a mean accuracy of 99% [11]. In addition, a recent meta-analysis revealed that ultrasound-guided hip joint injections were significantly more accurate than landmark-guided intra-articular hip injections (accuracies were 100%, 95%CI 98–100%; 72%, 95%CI 56–85%, respectively) [12].

To date, many trials examining efficacy of intra-articular corticosteroid injection for osteoarthritis, either under fluoroscopy [13,14] or ultrasound-guidance [15,16], have revealed short-term improvement of hip pain [17], though no reliable predictors of response to intra-articular corticosteroid injections have been identified [18]. While it has been shown that no significant difference in terms of effectiveness between fluoroscopy and ultrasound-guided corticosteroid hip injection [19], Byrd, et al. [20] reported that ultrasound-guided injections were less painful and preferred by patients.

Ultrasound-guided intra-articular hip injections with hyaluronic acid (HA) products [15] and PRP [21] have also been performed in patients with hip osteoarthritis. Migliore, et al. [22] conducted a large prospective cohort study of 1022 patients with a seven-year follow-up supported the clinical efficacy and safety of HA in patients with hip osteoarthritis. A recent meta-analysis, however, concluded that there was still a lack of standardisation of HA for hip conditions and proposed that this is the best conservative therapy before surgery [23]. Similarly, another systematic review supported the safety and benefit in PRP treatment for hip osteoarthritis but given the considerable heterogeneity between studies and cost, further research is needed to establish the optimal PRP protocol [24].

#### Injection approach

**Figure 2:**
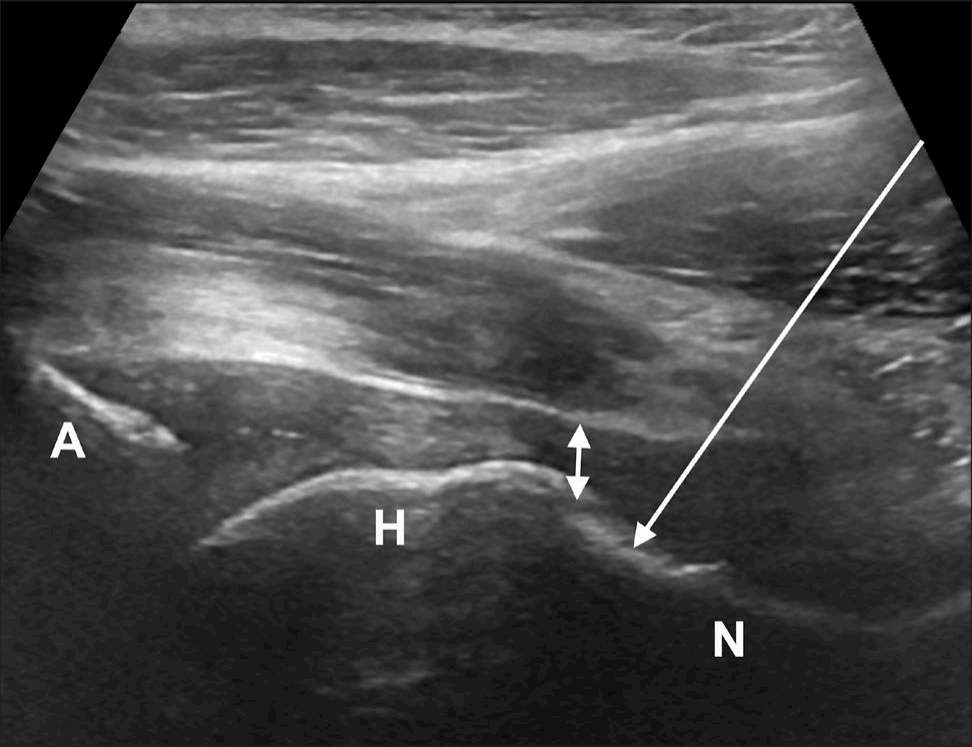
Anterior longitudinal view of the hip joint. The needle is introduced from an inferior and anterior approach, lateral to the femoral neurovascular bundle (arrow). A, acetabulum; H, femoral head; N, femoral neck; double arrow – anterior joint recess.

**Figure 3:**
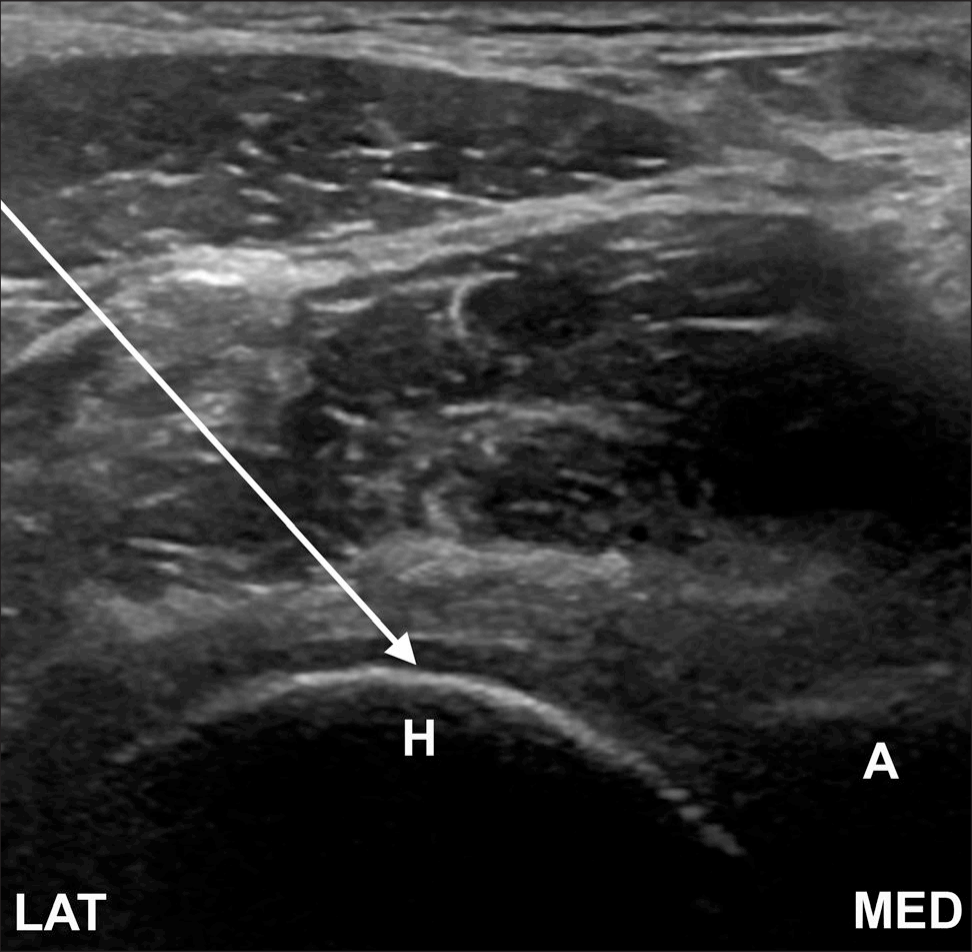
Transverse image of the hip joint. The needle is introduced from a lateral and anterior approach, to rest on the femoral head (arrow). A, acetabulum; H, femoral head; N, femoral neck; LAT, lateral; MED, medial.

Two common anterior approaches are typically used both with the patient lying supine. Many prefer the anterior longitudinal approach with the probe aligned along the long axis of the femoral neck. The needle is introduced from an anteroinferior approach and is passed into the anterior joint recess at the femoral head-neck junction (Figures 1 and 2). Another approach is with the ultrasound probe oriented axially and the femoral head and acetabular rim in view. This often shortens the distance from needle skin entry to joint compared to the longitudinal approach making it a useful approach in larger patients. The needle is introduced from an anterolateral approach, remaining lateral to the femoral neurovascular bundle, and the needle is advanced until its tip rests on the femoral head (Figure 3) [25]. The total volume injected is usually 6–7ml.

### Iliopsoas

Directly anterior to the hip joint is the iliopsoas muscle-tendon complex with the main tendon inserts on the lesser trochanter. The iliopsoas bursa is the largest bursa around the hip. It is located in between the iliopsoas muscle and hip joint, and may communicate with the hip joint in up to 15% of the population [26].

Disorders of the iliopsoas are a recognised significant source of hip or groin pain. Due to the close proximity of iliopsoas musculotendinous structures and bursa to the hip joint and acetabular rim, they are subjected to mechanical stress typically in the setting of overuse injury, acute trauma, or after total hip arthroplasty secondary to impingement by the prominent anterior rim of acetabular implant or the collar of the femoral prosthesis. Iliopsoas bursal distension is frequently complicated by hip joint pathology including rheumatoid arthritis, osteoarthritis and septic arthritis [27]. Other potential abnormalities are snapping hip syndrome (coxa saltans interna) [28].

The first-line treatment for iliopsoas disorders are usually conservative, consisting of activity modification, non-steroidal inflammatory drugs, and physical therapy. When conservative therapy fails, an iliopsoas bursal or peritendinous anaesthetic-corticosteroid injection can be considered. Ultrasound has an important role in dynamic assessment of the iliopsoas tendon and guiding a safe method of delivering therapeutic or diagnostic injections. The only potential immediate complication is transient femoral nerve palsy [29]. Complete or near complete relief of the symptoms confirms the diagnosis of iliopsoas impingement. These injections can also provide long-term relief in snapping hips and a favourable response to injection has been shown as a predictor good outcome after surgical release of iliopsoas tendon [30]. In addition, Adler et al. [31] reported 90% of patients with iliopsoas-related symptoms after hip arthroplasty achieved significant relief without the need for surgery. Ultrasound-guided percutaneous tenotomy for the treatment of iliopsoas impingement after total hip arthroplasty has been performed with positive results, however this was based on a single pilot case study [32].

#### Injection approach

**Figure 4:**
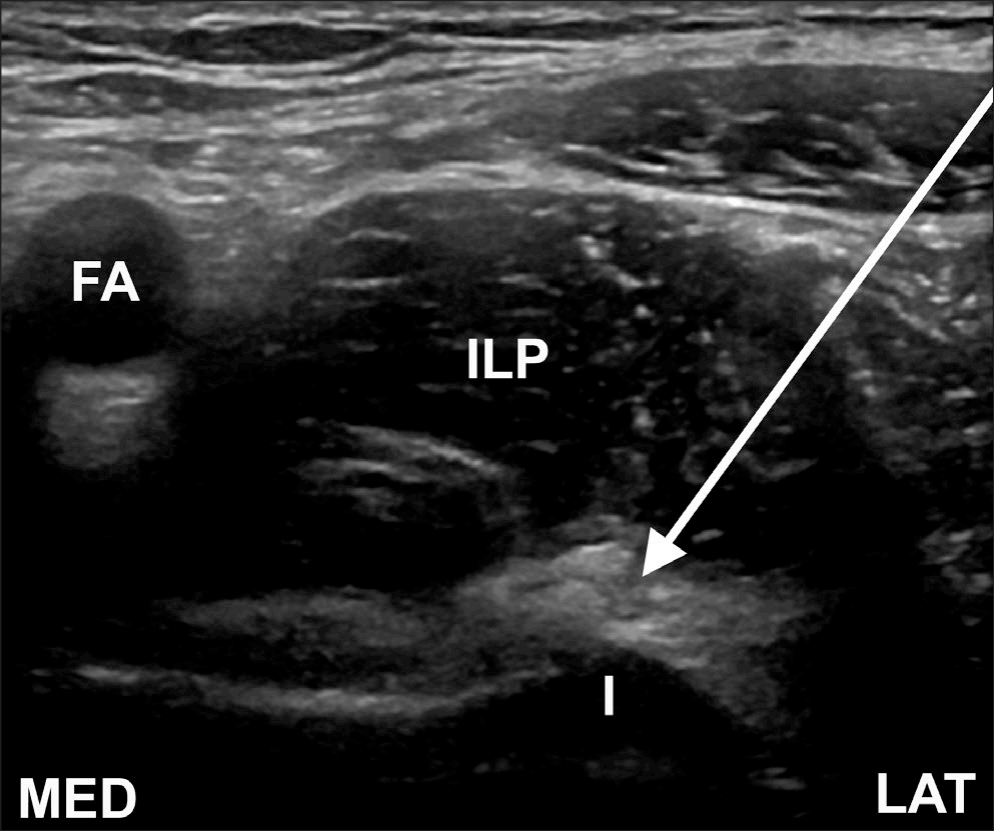
Transverse oblique ultrasound image superior to the femoral head, demonstrating the iliopsoas tendon. The needle is directed between the deep surface of the iliopsoas tendon and the superficial surface of the ilium (I) from a lateral approach (arrow) at the level of iliopectineal eminence. FA, femoral artery; ILP, iliopsoas muscle; LAT, lateral; MED, medial.

The patient is in supine position with the hip in neutral rotation. The transducer is placed transverse to the iliopsoas tendon, parallel to the inguinal ligament and the needle is inserted from a lateral to medial approach. The needle tip is positioned in between the iliopsoas muscle-tendon complex and the ilium at the level of the iliopectineal eminence (Figure 4) or alternatively between the iliopsoas tendon and acetabular rim [33]. Injectate volume is usually 7–8 ml [31].

### Greater trochanter

**Figure 5:**
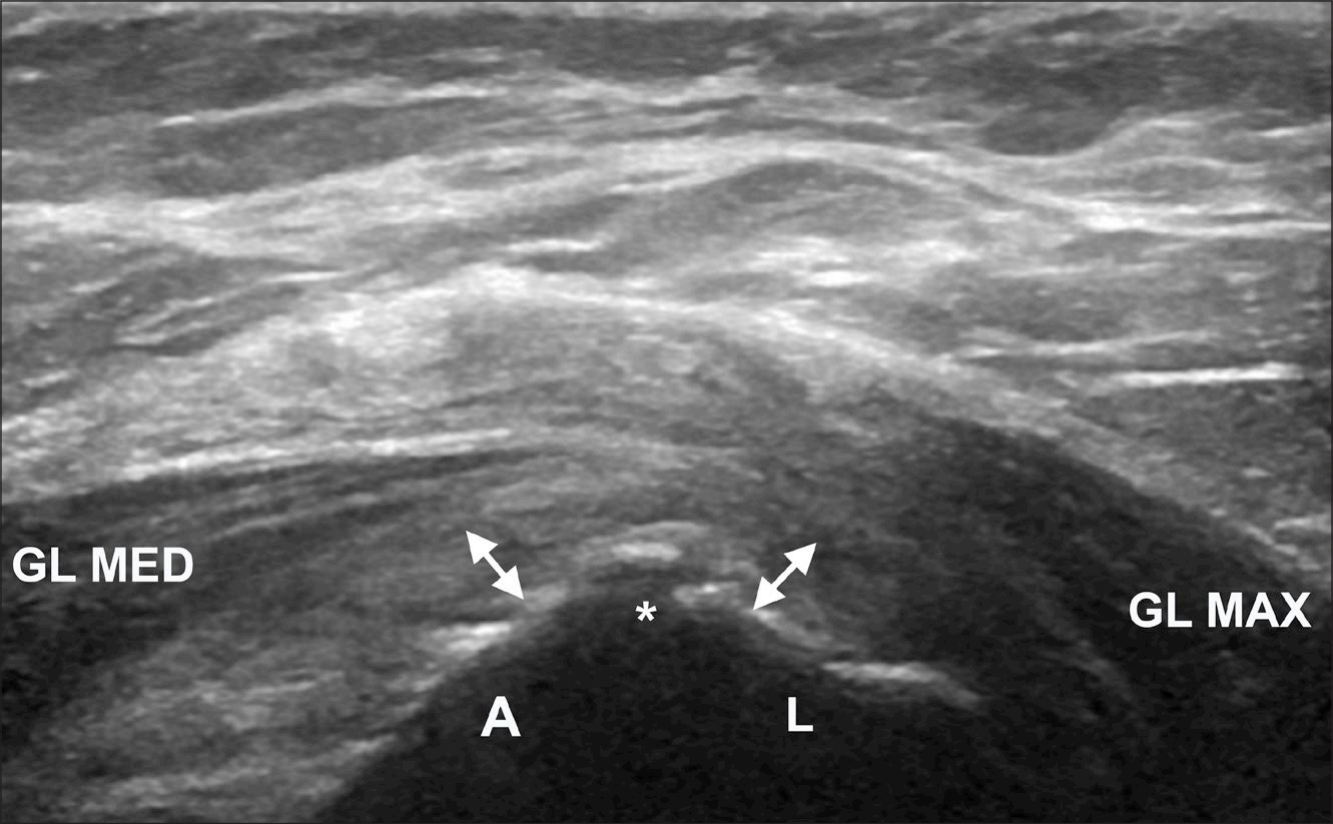
Transverse image over the greater trochanter showing the bony apex (asterisk) between the gluteus minimus tendon (double arrow) insertion onto the anterior facet (A) and the gluteus medius tendon (double arrow) insertion onto the lateral facet (L). GL MAX, gluteus maximus muscle; GL MED, gluteus medius muscle.

The greater trochanter arises from the lateral junction of the femoral neck and shaft, and has four distinct facets: anterior, lateral, posterior and superoposterior facets. The apex of the greater trochanter is seen between the anterior and lateral facets (Figure 5). The gluteus minimus is identified over the anterior facet, the gluteus medius over the lateral and superoposterior facets [34]. Superficial to the gluteus medius and minimus tendons is the fibromuscular sheath – iliotibial band, gluteus maximus posteriorly, and tensor fascia lata anteriorly. There are three bursae in the region – subgluteal minimus bursa is situated between the anterior facet and gluteus minimus tendon, the subgluteal medius bursa is in between the lateral facet and gluteus medius tendon, and the subgluteal maximus bursa, often referred to as trochanteric bursa, is lateral to the greater trochanter, in between the gluteal maximus and medius tendons [35].

Greater trochanteric pain syndrome (GTPS) is a relatively common condition found to affect 10–25% of the general population [36]. GTPS refers to pain originating from various structures in the lateral hip, which encompasses a number of disorders such as trochanteric bursitis, gluteal medius and minimus tendinopathy or tear and coxa saltans externa [37]. Most cases of GTPS (up to 60%) are self-limiting and tend to resolve with conservative management including activity modification, anti-inflammatory medication and physical therapy [38]. When these conservative measures fail, corticosteroid injections can be performed.

In a routine clinical setting, greater trochanteric injections are performed using landmarks without image-guidance. There is strong evidence of a short-term benefit with corticosteroid injection with most patients needing only a single injection, but up to 33% required a second injection and some as many as five injections [39,40]. A specific indication for image-guided injection is often for the treatment of patients who do not respond initially to a blind corticosteroid injection or patients with a large body habitus. Cohen, et al. [41] compared fluoroscopy-guided with blind injection in 65 patients with GTPS and found no significant differences in outcome at one month. Similarly, a recent RCT showed no significant differences between ultrasound-guided and blind injections at eight-week follow-up but patients receiving ultrasound-guided injection did perceive greater benefit [42]. Interestingly, McEvoy and colleagues [43] reported greater improvement with ultrasound-guided injection to the trochanteric bursa compared to the subgluteal bursa at two-week follow-up but numbers were small and further follow-up was not performed.

In contrary to a prior misconception that symptoms were due to bursitis, recent evidence points to tendinopathy as the possible underlying aetiology [36]. Jacobson, et al. [44] therefore proposed that treatment should be directed to the underlying tendon condition and showed both ultrasound-guided tendon fenestration and PRP injection were effective for treatment of gluteal tendinosis (71% and 79% improvement at 92 days, respectively) in a blinded prospective trial consisted of 30 patients. However, randomised-controlled trials (RCTs) comparing all these treatment options are necessary to further determine their role in treating GTPS.

#### Injection approach

**Figure 6:**
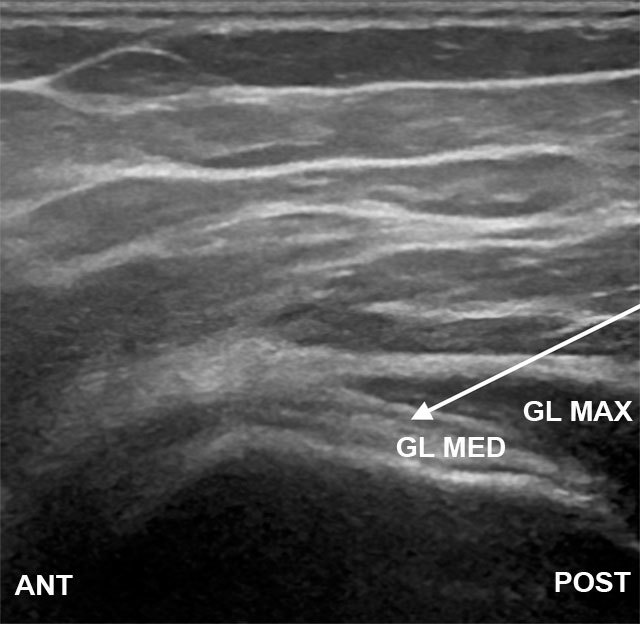
Transverse plane over the greater trochanter. The needle is advanced into the tissue plane between the gluteal maximus-iliotibial band and gluteus medius tendon from a posterior approach (arrow). GL MED, gluteus medius tendon; GL MAX, gluteus maximus muscle; ANT, anterior; POST, posterior.

Trochanteric bursa – The patient is positioned in the lateral decubitus position with the symptomatic hip facing upward and the hips and knees are flexed. The transducer is positioned in the anatomic traverse plane perpendicular to long axis of femur. The needle is introduced using a posterior to anterior approach and is passed into the interface of gluteus medius tendon and the gluteus maximus-iliotibial band (Figure 6) [33].

### Pubic Symphysis/Common Adductor-Rectus Abdominis Aponeurosis

**Figure 7:**
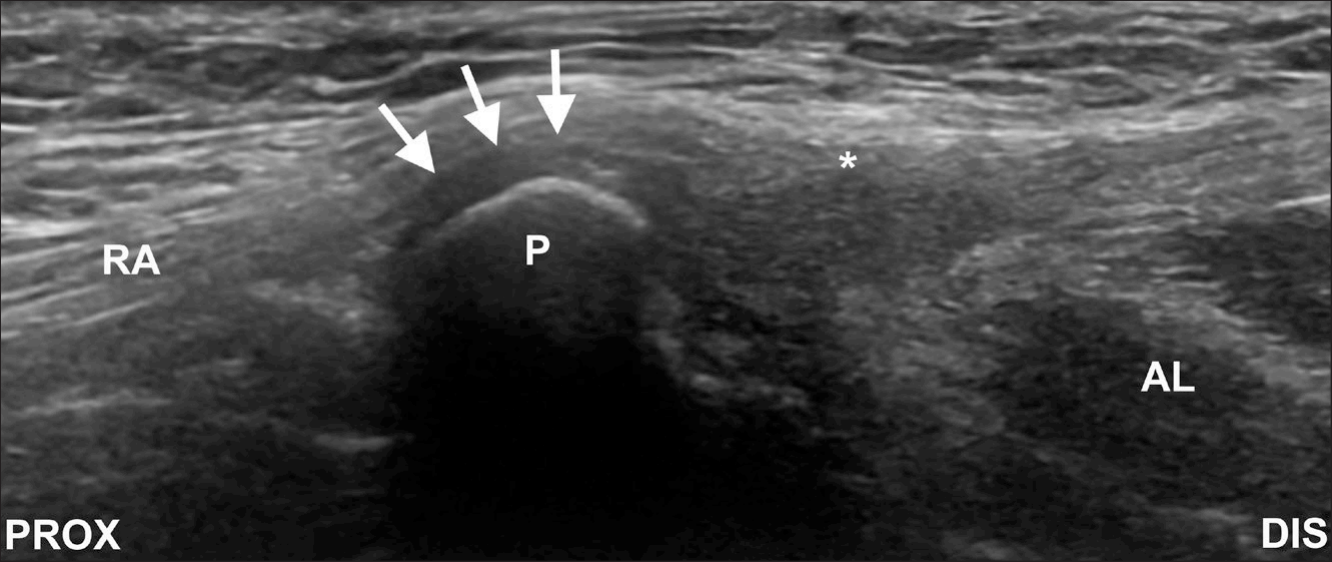
Sagittal image shows merging of the anterior capsular tissues (arrows), pyrimidalis, rectus abdominis (RA), adductor longus muscle (AL) and its tendon (asterisk). P, pubis; DIS, distal PROX; proximal.

The pubic symphysis is an amphiarthrodial joint composed of paired pubic bones with each articular surface covered by hyaline cartilage and separated by an intervening fibrocartilaginous disc with ligaments span the joint. It has limited mobility but stabilises the anterior pelvis during movements with numerous contributing musculotendinous attachments both directly onto its capsule but also onto the skeleton and soft tissues immediately adjacent to it. Of these, the most critical for maintaining the stability are rectus abdominis and adductor longus, which are contiguous and merge with the pubic symphysis capsular tissues in a rectus-abdominis-adductor aponeurosis (Figure 7) [45].

Athletic pubalgia, sports hernia, sportman’s hernia, Gilmore groin, adductor dysfunction or tendinopathy and osteitis pubis are various terms used to described entities that are either in the same spectrum of disease or share similar mechanism of injury and clinical manifestation. These are common sources of groin pain in athletes, especially those involved in single stance manoeuvres such as soccer, rugby and ice hockey.

Initial management of these conditions is usually conservative, including rest, core physical rehabilitation and non-steroidal anti-inflammatory drugs. In patients who fail to respond to conservative therapy, injection into the pubic symphysis or the entheseal soft tissue can be considered. Schilders, et al. [46,47] reported one year of pain relief was achieved following a single entheseal pubic cleft injection for adductor-related groin pain in 68% of 28 recreational athletes and in all of the seven competitive athletes with normal findings on MRI but 94% of 17 competitive athletes with enthesopathy confirmed on MRI had experienced a recurrence. Ultrasound-guided needle tenotomy, PRP injection and prolotherapy with dextrose and lidocaine injection have also been described with symptom improvement following treatment but these were only in a case report and small pilot study [48,49]. A systemic review in treatment of osteitis pubis in athletes identified only level four evidence with 24 case series in a total of 195 athletes without any direct comparison of treatment modalities [50]. The mean return to play was 9.6-weeks using conservative treatment, eight-weeks for corticosteroid injections, nine-weeks following prolotherapy [50].

#### Injection approach

**Figure 8:**
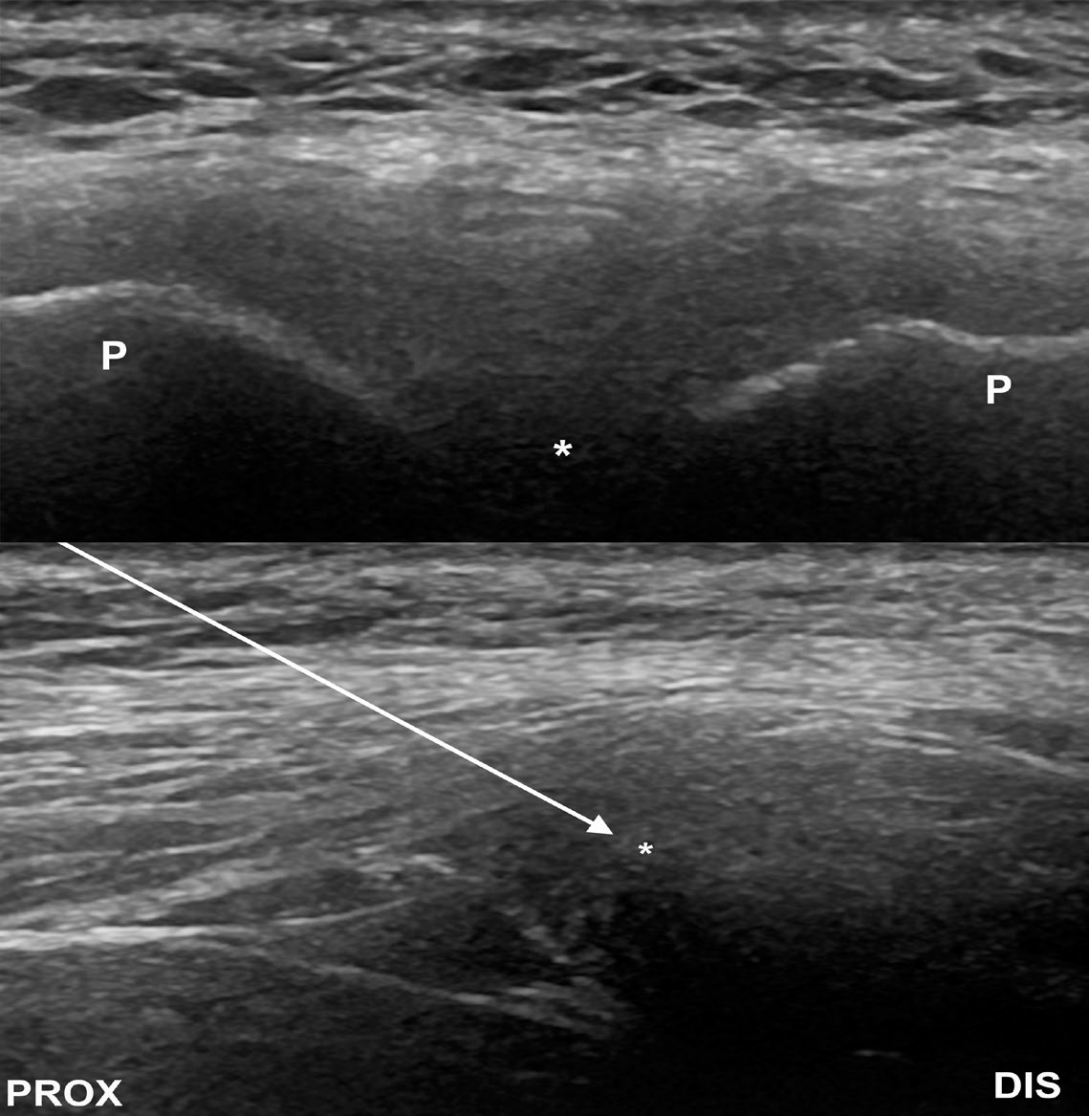
(A, image above) Transverse image of the symphysis pubis. P, pubis. (B, image below) Sagittal ultrasound image for symphyseal injection. The needle is introduced from a superior approach (arrow). Asterisk indicates the joint. DIS, distal; PROX, proximal.

Pubic symphysis – The patient is in supine position. The symphyseal joint space is initially scanned on the midline pubis in a transverse orientation (Figure 8A), the probe is then turned to the sagittal plane and centred over the anterior joint space. The needle is introduced from superior aspect of the probe and directed into the superior aspect of the joint and disc (Figure 8B). The medication injected depends on the indication for the procedure. Joint volume capacity is usually less than 2 ml.

Capsular/adductor entheseal soft tissue – The patient is in supine position with the leg slightly abducted and externally rotated at the hip. MRI and clinical findings are used for planning the site. The approach is usually lateral and inferior to the scrotum and the needle is advanced directly into the tissues [51]. Anaesthetic is injected into the soft tissues and is followed by injectate or dry needling.

### Lateral femoral cutaneous nerve (LFCN)

**Figure 9:**
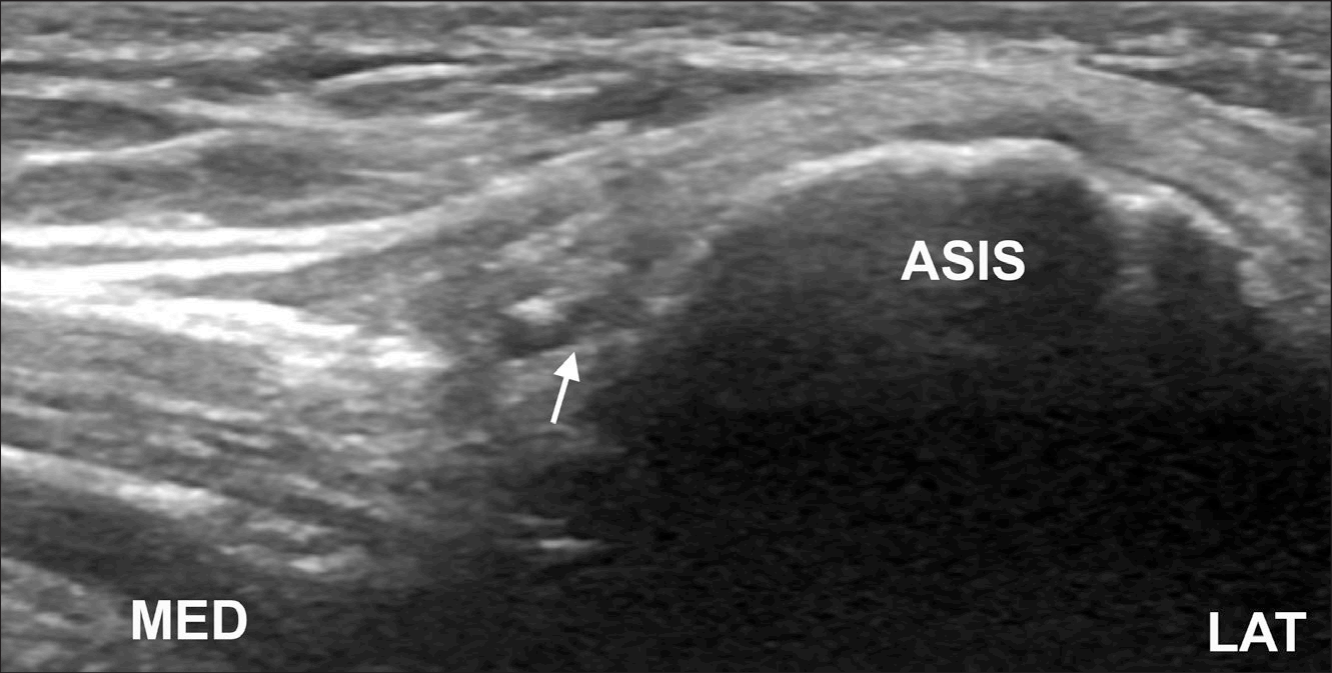
Tranverse oblique image shows LFCN (arrow) at the level of anterior superior iliac spine (ASIS). LAT, lateral; MED, medial.

The LFCN is primarily a sensory nerve and arises from the lumbar plexus, mainly deriving its fibres from L2 to L3 nerve roots. It then runs along the lateral border of psoas major, crosses the iliacus and passes through a fibrous tunnel formed by a small split in the lateral end of the inguinal ligament, medial and inferior to the anterior superior iliac spine (ASIS) (Figure 9). The nerve then passes under the inguinal ligament and over the sartorius muscle and enters the thigh as it divides into anterior and posterior branches that supply the anterolateral and lateral aspects of the thigh [52]. Several different anatomic variations in its course have been described [53].

Meralgia paraesthetica (MP) is an entrapment neuropathy of LFCN, resulting in pain, paraesthesia and sensory loss over the anterolateral aspect of the proximal thigh. This is commonly caused by entrapment at the level of inguinal ligament and is usually in association with obesity, pregnancy and ascites [54]. Other causes include, external compression by belts, leg-length discrepancies, iatrogenic (bone graft harvesting), pelvic and retroperitoneal tumours, neuropathy associated with diabetes or AIDS [54]. The occurrence of MP in various sports including gymnastics, soccer, bodybuilding and baseball have also been observed [55].

Treatment is usually conservative which includes avoidance of traumatic or external compressive factors, and surgical decompression may be considered in patients who do not respond. Injection of local anaesthetic with corticosteroid at the presumed site of entrapment at the inguinal ligament may offer an alternative to surgery and may also have diagnostic value. The injection of the LFCN is classically described using anatomical landmarks but the failure rates have been reported to be as high as 60% [56]. Ultrasound-guidance is useful in facilitating perineural injection and is more accurate than anatomical landmarks, particularly in patients with anatomical variation of the LFCN [57,58]. However, there is no RCT assessment of efficacy of treatments for MP. A recent systematic review identified only four high-quality observational studies which reported high improvement rates of 83% following local injection of corticosteroid in a combined total of 157 cases [54]. The paucity of research on this subject has therefore made it a challenge to diagnose and treat this condition.

#### Injection approach

**Figure 10:**
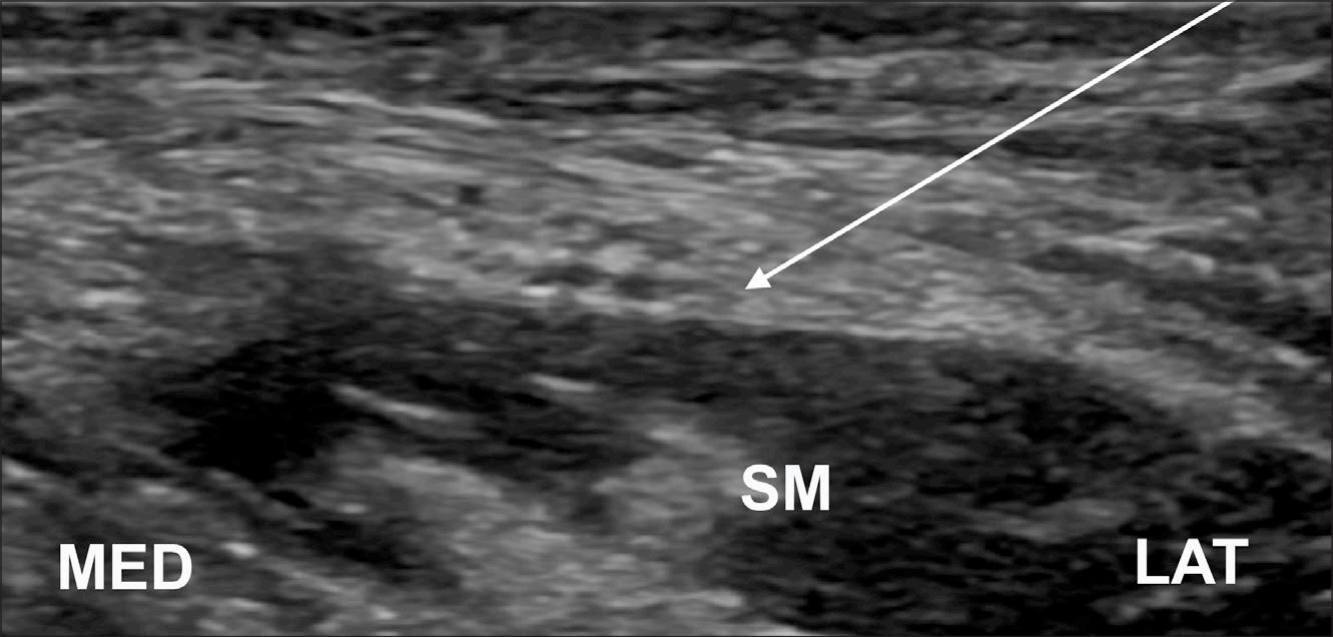
The needle is introduced from a lateral approach (arrow) for LFCN perineural injection. SM, sartorius muscle; LAT, lateral; MED, medial.

The patient is in supine position. The lateral end of the probe is placed on the ASIS in an anatomic transverse plane and the medial part of the probe is angled in a caudal direction, so the transducer is parallel with the inguinal ligament. The probe is subsequently moved in a medial-caudal direction to search for LFCN, an oval hyperechoic structure containing several circular hypoechoic fascicles. The needle is inserted in plane from a lateral to medial approach for perineural injection (Figure 10) [58]. Injectate volume is usually 7–9 ml [57,58].

## Conclusion

Various ultrasound-guided diagnostic and therapeutic injections can be considered in patients with hip or groin pain. Many studies have demonstrated the safety, accuracy and efficacy of these techniques, although there is lack of standardisation regarding the injectates used and its long-term benefit remains uncertain. Given the advantages of portability, lack of ionising radiation, and visualisation of soft tissue and neurovascular structures, ultrasound-guidance is a highly practical and recommended technique when performing injections in the hip and groin region.
